# Investigating Child Abuse in Sports: An Ecological Systems Perspective

**DOI:** 10.3390/children11121487

**Published:** 2024-12-06

**Authors:** Damla Güler, Yağmur Güler, Caner Cengiz, Semiyha Tuncel, Raci Karayiğit

**Affiliations:** 1 Department of Physical Education and Sports, Faculty of Sport Sciences, Ankara University, Ankara 06830, Turkey; gulerd@ankara.edu.tr (D.G.); canercengiz@ankara.edu.tr (C.C.); semiyha.dolasir.tuncel@ankara.edu.tr (S.T.); 2 Department of Recreation, Faculty of Sport Sciences, Yalova University, Yalova 77200, Turkey; yagmur.guler@yalova.edu.tr; 3 Department of Coaching Education, Faculty of Sport Sciences, Ankara University, Ankara 06830, Turkey

**Keywords:** child abuse in sport, ecological systems, physical, emotional and sexual abuse, neglect

## Abstract

Background: This study examines child abuse in sports environments through Ecological Systems Theory, revealing the multifaceted nature of abuse and the impact of environmental factors at various levels. Methods: With a study design using the phenomenology approach, a qualitative research method, data were collected through semi-structured, in-depth interviews with 11 Turkish participants, including 5 athletes, 4 coaches, and 2 academics with coaching experience in Sports Sciences. Thematic analysis was used to evaluate the data, categorizing findings into four levels: microsystem, mesosystem, exosystem, and macrosystem. Results: Findings show that at the microsystem level, children are exposed to physical, emotional, and sexual abuse, as well as neglect, largely through interactions with coaches and teammates. Physical abuse includes non-contact forms, like dehydration and forced training, and contact abuse, such as violence from coaches and peers. Emotional abuse manifests through psychological pressures and verbal attacks, creating a toxic environment. At the mesosystem level, excessive parental trust in coaches reduces oversight, leaving children vulnerable. In the exosystem, inadequate supervision of coaches and a lack of response to abuse cases by sports clubs worsen the issue. At the macrosystem level, cultural norms and societal attitudes normalize abuse, especially affecting female athletes. Conclusions: The study highlights the need for comprehensive interventions, including family awareness, stricter coach oversight, and robust policies within sports organizations to protect children. By emphasizing the interaction of individual, familial, and societal factors, this research underscores the importance of collective efforts to ensure safer sports environments.

## 1. Introduction

Child abuse is a painful reality that continues to exist in the world of sports, as in many areas of life. Abuse is defined as a pattern of behavior involving actual or potential harm committed by a caregiver (e.g., parent, coach) toward an athlete, which may result in physical, sexual, emotional, or neglectful mistreatment [1]. Abuse in sports occurs in four main forms: physical abuse, emotional abuse, sexual abuse, and neglect [1,2]. Abuse leads to a number of negative symptoms such as low mood, self-esteem, self-efficacy, leaving the sport [3,4], poor performance [5], eating disorders [6,7] anxiety, depression [8], drug use, and suicide attempts [9]. and negatively affects the mental health and quality of life of athletes in adulthood [10]. Additionally, there is a positive and statistically significant association between childhood adverse experiences (abuse and types of abuse, neglect and types of neglect, teasing, and bullying) and body dysmorphic disorder symptomatology, except for sexual abuse. This suggests that higher levels of childhood adverse experiences are associated with higher levels of body dysmorphic disorder symptomatology in individuals [11].

Child abuse in sport is a common and frequent experience for children in different national contexts such as Austria, Belgium (Brussels-Wallonia and Flanders), Germany, Romania, Spain, and the United Kingdom. The European Statistics (CASES) project determined prevalence rates for five categories of violence against children in sport: physical, psychological, non-contact sexual, contact sexual, and neglect, based on one or more experiences of interpersonal violence (IVAC) before the age of 18 among respondents aged 18–30. According to the findings, the rate of psychological violence was found to be up to 65%, physical violence was 44%, neglect was 37%, non-contact sexual violence was 35%, and contact sexual violence was 20%. Although national differences exist, IVAC rates have been found to be broadly similar across countries [12]. Studies conducted in Turkey have also revealed the existence of child abuse in the field of sports [13,14,15,16].

Factors that increase the risk of athlete mistreatment include insufficient oversight in accessing young athletes, inadequate training of sport stakeholders, inadequate policies and codes of conduct, overemphasis on performance, and the self-regulatory nature of sports [17]. Failures to prevent abuse in sports are largely due to interventions that target only individuals or cases and ignore organizational or ecosystem-level factors [18,19]. Although individual factors play an important role in explaining abuse, it is stated that abuse does not only occur due to individual reasons but rather is a result of interactions between individuals, the organizational environment, and broader social factors [3]. Ref. [20] reviews current initiatives to prevent maltreatment in sports, highlighting protection gaps in this area and highlighting the need for interventions at different levels (micro, meso, exo and macro) to improve athlete well-being. Additionally, prevention and intervention programs coordinated by sports organizations should encompass all abusive and violent behaviors toward child athletes rather than focusing on just one type of abuse (e.g., sexual abuse and exploitation) [10]. Child abuse in sports environments has often been addressed in terms of a specific dimension (e.g., physical, emotional, or sexual abuse) or a specific level (e.g., the coach–child relationship). Within the framework of Ecological Systems Theory, studies examining child abuse in all its aspects and at different levels (microsystem, mesosystem, exosystem, smf macrosystem) are limited [21,22,23]. There is a striking lack of research in the literature that adopts this comprehensive approach. This study aims to fill an important gap in this field by addressing child abuse in sports environments in a multidimensional and systematic way. In this context, the aim of the study is to examine child abuse in sports environments within the framework of Ecological Systems Theory and to determine what role environmental factors play in this process.

This study is one of the few studies that adopt Bronfenbrenner’s Ecological Systems Theory to comprehensively address child abuse in sports environments, covering the microsystem, mesosystem, exosystem, and macrosystem levels. Previous studies in the literature have generally focused on specific levels (e.g., coach–athlete interactions) but have not examined abuse in the context of broader environmental and sociocultural factors. Our study makes a significant contribution to the literature by addressing the interconnected factors contributing to child abuse from a holistic perspective. We also emphasize that interventions aimed at preventing abuse should be shaped by considering risks and opportunities at each level.

In this context, our study provides findings that will guide protective interventions in line with the risks identified at each level, thus paving the way for the development of a multi-layered and holistic approach to preventing abuse. In this respect, our research demonstrates that child protection strategies in sports environments should be addressed from a broad ecological perspective, not only through individual or limited interactions.

### Ecological Systems Theory

The ecology of human development involves the scientific study of the process of mutual adaptation between an actively growing human and the changing characteristics of the environment in which she/he lives. This process is affected by changes in the environments in which the developing individual lives, as well as by the relationships between these environments and broader environmental factors [24].

Bronfenbrenner’s Ecological Systems Theory is an approach that explains the development and socialization of an individual through different width circles or layers of the environment with which they interact. According to this theory, the individual both affects and is affected by their environment. The environment provides conditions and constraints that direct the individual’s development. The environment consists of four main interconnected systems: microsystem, mesosystem, ecosystem, and macrosystem [24]. The microsystem refers to the individual’s immediate environment; the mesosystem refers to the interactions between these environments; the exosystem refers to the environment that the individual is indirectly affected by; and the macrosystem refers to the broad cultural and social structure in which the individual is located. The relationships between these systems shape the individual’s development and socialization process [25]. Bronfenbrenner’s Ecological Systems Theory has provided a framework for many issues concerning public health, such as the prevention of child abuse [26], sexual violence in sports environments [27], and problems facing social psychology [28].

## 2. Materials and Methods

In this study, a phenomenological research design, one of the qualitative research designs, was used to understand child abuse in sports environments and the role of environmental factors in this process. A phenomenological study identifies the shared meaning of several individuals’ experiences of a concept or phenomenon. A phenomenology provides an in-depth understanding of a phenomenon as experienced by several people [29]. In this context, this study aimed to examine in depth the experiences of child abuse in sports environments.

### 2.1. Ethical Considerations

Given the sensitivity of the topic, several ethical safeguards were implemented to ensure the protection of participants’ rights, privacy, and emotional well-being.

Ethics Approval and Informed Consent: Ethical approval for this study was obtained from the Human Research Ethics Committee of Bartın University (Decision No: 2023-SBB-0523). During the informed consent process, participants were provided with detailed information about the study’s purpose, voluntary participation, confidentiality of data, and their right to withdraw from the study at any time. This ensured that participants were fully informed and agreed to the conditions of the research before their involvement.

Confidentiality and Privacy Protection: Considering the sensitive nature of the information shared, rigorous measures were taken to protect participants’ identities. All data were anonymized to safeguard participants’ privacy, with personal identifiers removed and pseudonyms used in reporting. Interview records were securely stored and accessed only for analysis purposes, ensuring that participants’ identities remained confidential.

Emotional Support and Safety: Given the potentially distressing nature of discussing experiences of child abuse, interviews were conducted in a manner that prioritized participants’ emotional comfort. Participants were informed that they could pause or end the interview at any time.

Researcher Objectivity and Sensitivity: The research team maintained a neutral stance to understand participants’ experiences objectively. Interviews were conducted with a high level of empathy and cultural sensitivity, allowing participants to openly express their experiences and perceptions. This approach helped minimize researcher bias and ensured that participants’ accounts were respected and accurately represented.

### 2.2. Participants

Within the scope of this study, the participants consisted of a total of 11 people, including 5 athletes, 4 coaches, and 2 academics who specialize in the field of Sports Sciences and have coaching experience. Participants were selected using a purposive sampling method, focusing on individuals who play various roles in sports environments and have direct or indirect experiences with child abuse or neglect in these settings. This purposive sampling approach supports the aim of obtaining insights into different dimensions of the sports environment and examining abuse in sports from a multifaceted perspective. The researchers identified these participants through personal contacts among individuals residing in various provinces of Turkey. Although a formal preliminary survey was not conducted, an informal verbal assessment was carried out during initial contact with each participant to better understand their level of experience related to abuse in sports. These preliminary conversations provided insights into each participant’s perspective, helping to select individuals who could make meaningful contributions to the study. Participants were selected using a purposive sampling method, focusing on the experiences of individuals who play roles at different levels in sports environments. The researchers identified these participants through personal contacts among individuals residing in various provinces of Turkey. Preliminary interviews with these individuals were conducted face-to-face and by telephone, and the participants were given detailed information about the subject and purpose of the study. In this process, the researchers identified which participants had experienced abuse, and these individuals were included in the participant group after they accepted the meeting requests. The athletes among the participants specialize in rhythmic gymnastics, football, hockey (2), and volleyball. These athletes are between the ages of 22 and 27 and have 10 to 18 years of sports experience. The coaches are experts in volleyball (2), football, and karate and have 8 to 17 years of coaching experience. Coaches’ ages range from 27 to 48. Academics are people who specialize in Sports Sciences and also have coaching experience. During this study, athletes shared their experiences up to the age of 18. Coaches and academics described their experiences with children in the sports environment. To protect the confidentiality of the participants, pseudonyms were used instead of their real names.

### 2.3. Data Collection

Data in the study were collected through semi-structured interviews consisting of open-ended questions. Open-ended questions used in semi-structured interviews allow participants to convey their perceptions through their thoughts [30]. For this reason, open-ended questions were used in the interviews. In order to ensure the validity of the semi-structured interview form prepared in line with the research questions, taking into account the Ecological System Theory, expert opinion was obtained. The interview form was reviewed by a panel of 3 experts, including (a) measurement and evaluation specialist(s) and researchers specializing in child protection. These experts provided feedback on the clarity, relevance, and comprehensiveness of the questions, ensuring alignment with the study’s objectives and theoretical framework. Based on this feedback, necessary revisions were made before implementing the preliminary application. The interview guide included open-ended questions designed to elicit detailed responses related to the types, causes, and environmental factors associated with abuse. Sample questions included “Can you share a specific situation where you have witnessed or experienced neglect and abuse in sports settings? What are your views on environmental factors that increase the risk of neglect and abuse in sports environments”?

The interviews were conducted face-to-face using the ZOOM platform. These interviews lasted between 20 and 80 min. The audio recordings of the interviews were transcribed into written text by the researcher. At the end of this process, a total of 150 pages of text were obtained from all interviews.

### 2.4. Data Analysis

Qualitative analysis of the collected data was performed using thematic analysis. A theoretical thematic analysis tends to be driven by the researcher’s theoretical or analytical interest in the field and is therefore more explicitly analyst-focused. This form of thematic analysis tends to provide a detailed analysis of some aspect of the data rather than a rich description of the data overall [31].

In this study, the outline of the thematic analysis method by Braun and Clarke [30] was used. Analysis with a theoretical approach requires interaction with the literature in the first stage. In this study, the theoretical thematic analysis method was used. First, familiarity with the data was gained based on the theory determined in line with the research question, and initial codes were created. The coding process involved systematically coding interesting and meaningful aspects of the data in accordance with the theoretical framework. The codes obtained were grouped around predetermined theoretical themes. The suitability of the themes for both the coded data and the general data set was reviewed, and an analysis map was created based on these themes and the theoretical framework. Finally, in the process of identifying and naming themes, the characteristics of each theme were clarified based on the theoretical framework. They were categorized according to the four levels of Ecological Systems Theory (microsystem, mesosystem, exosystem, macrosystem), and the types of child abuse that occur and the environmental factors that affect the emergence of this situation were determined. To increase the robustness of the analysis, two separate researchers participated in coding and analyzing the responses. To ensure consistency, the experts conducted independent coding followed by a consensus meeting where discrepancies were discussed and resolved.

## 3. Results

The findings reveal child abuse in sports environments and the role played by environmental factors in this process within the framework of Ecological Systems Theory. In line with the participants’ opinions, the findings were discussed in detail at the microsystem, mesosystem, exosystem, and macrosystem levels.

### 3.1. Microsystem-Level Findings

Microsystem refers to the environments with which the child directly interacts. At this level, the types of abuse that children are exposed to in their interactions with their coaches, teammates, and other athletes are examined.

#### 3.1.1. Physical Abuse

Participants expressed physical abuse as contact and non-contact. As non-contact physical abuse, participants stated that children were frequently dehydrated and encouraged to use doping and steroids. It has also been stated that injured athletes are forced to continue playing and are forced to train on unsuitable grounds.

For example, one coach described how athletes are forced to continue when injured as follows:

“The motto of ‘no pain, no gain’ is a motto that causes us trouble. There is no pain, there is winning, I don’t have to suffer. One day, an athlete sprained foot during a match. The doctor said it could continue… The athlete is not asked because the doctor says he or she can continue. Do you want to continue? Are u okey? The injury that would have healed in perhaps a week continues for a month and turns into a sprain that keeps recurring. I have experienced and seen this”.

(Ayşe, Coach)

An athlete who stated that training on unsuitable ground was challenging shared her experience as follows:

“The pitch is very bad, it is no different than concrete, but we train on that pitch for 2–2.5 h. We do running training. This is also a kind of strain because then there is no muscle or leg left, everywhere is filled with edema, lactic acid”.

(Seda, Athlete)

A coach who emphasized that doping and steroid use is a serious abuse said the following:

“Doping is a great example of abuse. There are coaches who give steroids, especially in athletics. There are trainers who perform butterfly intravenous access. There are trainers who have evolved into this field. We have experienced many of these with coaches in the past. The child’s blood levels are not important. They being in developmental age is not important. There are trainers who say they can take this supplement, eat that. This is very serious abuse”.

(Yavuz, Trainer)

Contact physical abuse includes physical attacks and violence that children are exposed to during their athletic careers. Participants described physical violence from teammates and coaches.

For example, a coach explained how he was subjected to physical violence by his teammates during his time as an athlete:

“My teammate says, ‘You did this in training, didn’t I warn you?’ 3–4 people come towards me and try to put pressure on me, beat me, push me, try to slap me. These kinds of situations can happen, unfortunately. I have been exposed to them during my time doing sports”.

(Ali, Coach)

One athlete explained that coaches in his branch used violence against athletes in the locker room:

“Since our branch is a very tough branch, the matches are fierce. During half-time, the girls were taken to the locker room and beaten by the coach. Even if the score of the match was ahead, they were using violence to ask why you made these mistakes in the match”.

(Buket, Athlete)

#### 3.1.2. Emotional Abuse

Emotional abuse refers to the psychological pressure and emotionally damaging behaviors that athletes are subjected to by their coaches or teammates. Participants reported being subjected to emotional abuse, including physical actions, social and psychological pressure, and verbal statements.

As for emotional abuse involving physical actions, participants stated that behaviors such as throwing objects in anger and breaking doors in the locker room were common.

For example, one athlete described how her coaches became angry when she made mistakes:

“He would punch the benches during the match when we made mistakes. We would feel very bad”.

(Seda, Athlete)

Emotional abuse, which includes social and psychological pressure, refers to situations where athletes are forced to participate in matches and training, are motivated through fear, and are kept under excessive control. Participants stated that their coaches tried to motivate athletes through fear by constantly pressuring them.

For example, an athlete described the pressure she experienced before training as follows:

“12 h before our training, our coach would make us stand up and constantly scold us. He was yelling and cursing at us. We were playing for fear of him and we were successful because of his fear”.

(Seda, Athlete)

Another athlete stated that it has become a rule to participate in training by force, even in cases of pain and injury:

“The player has a pain somewhere and cannot train. But the mentality we were taught was, even if you have a fever, you will go out for that match, even if it hurts, you will do it. There is unnecessary pressure, like you will play as long as you don’t die”.

(Hülya, Athlete)

Peer bullying also creates serious pressure on athletes and negatively affects their performance. An academic expressed that small athletes are hesitant to surpass big athletes as follows:

“There was peer bullying. Small athletes were making mistakes on purpose so as not to surpass the big athletes. Because they knew the psychological pressure they would experience if they won”.

(Serkan, Academic)

Verbal emotional abuse includes situations where coaches and teammates use sexist language, insults, and derogatory comments toward athletes.

Participants reported that coaches and teammates belittled athletes by using sexist language and making derogatory comments:

“The coaches tell the girls, ‘You’re here to model‘. They were belittling them, saying they were playing like girls. They were treating women in a sexist manner. They were saying, “Your butts are playing separately.” I have witnessed such things”.

(Sude, Athlete)

One athlete stated that she was subjected to peer bullying by being made fun of for her clothes:

“Peers also make fun of them financially. For example, children can experience peer bullying such as ‘Are you still wearing this? Don’t you have any other workout clothes’?”.

(Didem, Athlete)

#### 3.1.3. Sexual Abuse

Sexual abuse was examined as non-contact and contact. Non-contact sexual harassment includes situations such as inappropriate comments and behaviours, and requests for inappropriate photographs. This type of abuse refers to sexual harassment and abusive behavior that athletes are subjected to without physical contact.

For example, an athlete described how her friend was sexually harassed by her coach at another club:

“A friend of mine was harassed by a trainer for whom I would call a pervert. His trainer would call him to his room, saying how nice his tights were, and lock the door”.

(Hülya, Athlete)

Another athlete stated that her coach asked her for inappropriate photographs:

“While I was talking to my coach, he suddenly asked me to send a photo. I was very surprised”.

(Seda, Athlete)

One coach described an incident where a young girl’s photograph was taken inappropriately:

“A boxing trainer’s gym was recently stoned in Ankara. He was caught taking pictures of a naked 14-year-old girl. “The weight was dropping, I was weighing it, that’s why I pulled it off,” he said.

(Yavuz, Coach)

A coach described the inappropriate behavior in the locker room as follows:

“This type of abuse can happen during adolescence. Those who take their clothes off and go into the shower, those who show various parts of their bodies. That’s why I’m in favor of keeping the age range very close when planning training. There should be same age groups in the locker room”.

(Ali, Coach)

Contact sexual abuse is defined as coaches touching athletes inappropriately. This type of abuse refers to sexual harassment that athletes are subjected to through physical contact.

An academic stated that it was disturbing when coaches touched athletes unnecessarily:

“Our old coach was a very fatherly person, but he gesticulated a lot. For example, it is not right to hug, touch or push someone even if there is no number”.

(Serkan, Academic)

Another academic stated that it could be uncomfortable for volleyball players to touch each other’s hips after each point:

“It feels strange when volleyball players touch each other’s hips after every point. Could it cause different discomforts? This move is made every time a point is lost or won. This doesn’t seem right to me”.

(Serkan, Academic)

#### 3.1.4. Neglect

Health and nutrition neglect includes situations such as athletes not following their weight control and diet and lack of health services. Such negligence can have serious negative effects on athletes’ physical and performance capacities.

For example, a coach expressed how athletes are neglected in their weight loss process as follows:

“Coach says you are controlling your weight, right? You will compete in 75 kilos. …. He says, ‘I’ll lose 3 kilos’, and for the last 10 days he has been trying to lose weight by starving himself, drinking only water, and running. During this process, he becomes incredibly exhausted, and his performance is affected by more than 50%. The coach should have checked the athlete by weighing him every day”.

(Yavuz, Coach)

An athlete expressed the security vulnerability created by the absence of medical personnel on the field as follows:

“Our risk of getting injured on the field is a situation that should be foreseen. The lack of medical personnel when injured is a security vulnerability. I may have a heart attack on the field, but there is no one with first aid knowledge”.

(Sude, Athlete)

An academic stated that the nutrition specialist ignored the needs of athletes and said

“The nutrition specialist was constantly referring to athletes as ‘patients’ and did not prepare a suitable nutrition program. Although the athletes trained for 7 h a day, appropriate meals were not prepared. The athletes could not show the desired performance”.

(Serkan, Academic)

Emotional neglect includes situations such as ignoring peer bullying, not providing emotional support, and acting indifferently. Such neglect can have serious negative effects on the emotional and psychological health of athletes.

One athlete stated that her coaches ignored her peer bullying as follows:

“The coach was ignoring the peer bullying. My teammates were jealous and ostracized me. The coach saw this and did nothing. The team members were tripping me, making me a goalkeeper, and throwing hard balls at me, and the coach was not intervening in any way. I felt defenseless and helpless”.

(Didem, Athlete)

Another coach stated that some athletes receive less attention than others, which could be considered emotional neglect:

“I have an athlete who does cycling. She says that her coach pays more attention to other students but is indifferent to her, and that this situation creates a feeling of inadequacy and loss of self-confidence”.

(Ali, Coach)

### 3.2. Mesosystem-Level Findings

The mesosystem includes the interactions between microsystems. At this level, the effects of the relationships between the family and the coach on child abuse are discussed.

#### Family and Coach Relationships

Participants stated that families give full authority to coaches and do not interfere excessively with their children’s sports activities. This situation can leave children vulnerable to abuse by coaches:

“I have seen many people say things like this to the other party, “Parent, the child is entrusted to you, to the trainer. Work your ass off, make him a good boxer, do whatever is necessary.” I think children of such parents are more vulnerable to abuse. After the kid heard something like that, I think the coach did something and didn’t tell his father. After this attitude of the family, the coach can treat the child as he/she wishes. This is a very dangerous attitude”.

(Serkan, Academic)

Families’ excessive trust in coaches in the sports environment and their unquestioning acceptance of what coaches say increases the risk of children being abused. One athlete stated that her family accepted what the coaches said without questioning and that this left the children vulnerable to abuse:

“When the coach would come and hit us on the head, knee, or foot, or pull our hair to throw us out of training, we would come and apologize to the coach. Usually, if the family became aware of this or if the family went and talked about it, they would explain that we are doing it for the good of the child, it has to be like this, he cannot develop otherwise, we cannot teach him otherwise, he has to receive this discipline, and the family would think, okay, so it has to be like this. So they accepted it by saying that this is how things are going”.

(Didem, Athlete)

Not allowing families to watch their children train results in the abuse that children are exposed to in sports environments going unnoticed. Participants stated that the problems experienced by children could not be recognized and intervened with because families did not participate in the training:

“The coach did not allow our families to be with us during training. This caused the problems we experienced to go unnoticed. “Nobody can see the bad behavior of the coaches or the bullying of our friends and we cannot share this with anyone”.

(Seda, Athlete)

### 3.3. Exosystem-Level Findings

The exosystem includes environments that the child does not interact with directly but that affect him/her indirectly. At this level, the management policies of sports clubs were examined.

#### Policies of Sports Clubs

Participants stated that coaches in sports clubs are subject to inadequate supervision and control mechanisms. This increases the risk of children being abused by malicious or incompetent coaches:

“If the coaches’ intentions are bad, certain sanctions against these people will prevent things that will happen. Well, that’s why it should be controlled a little more, maybe by the federation or if it were very widespread and known by everyone”.

(Sude, Athlete)

There have been statements that clubs do not take abuse cases seriously and do not apply the necessary sanctions:

“For example, my friend’s trainer wanted to experience something with her. Because she didn’t accept it, she made my friend sit on the bench for a season. She didn’t play at all, or her coach would send her to warm up and say from the first minute, ‘let that girl go and warm up for 90 min’, and then she would come back and sit on the bench. The club manager ignored this situation. “As long as the coach doesn’t get to the point of rape, as long as he doesn’t do something big, no one touches him”.

(Seda, Athlete)

### 3.4. Macrosystem-Level Findings

The macrosystem encompasses broader environmental factors such as cultural norms, values, and general belief systems. At this level, the social environment, cultural factors, policies, and procedures affecting child abuse were examined.

#### 3.4.1. Factors Originating from Social Environment and Culture

Participants stated that the way children are raised and the limited social environment increase the risk of children being abused in sports environments. Particularly in regions where children have limited social opportunities, the lack of opportunities for socialization and self-development makes children vulnerable to abuse:

“The child’s lack of opportunities, in other words, the lack of anything to do and the way they were raised, the lack of an environment that would allow children to socialize and develop themselves, caused them to be subjected to peer abuse when they went to a place with opportunities. For example, in the camp in the center of Rize, the children here had much more opportunities than those coming from outside. That’s why children coming from outside were not welcomed, they were bullied and made fun of”.

(Serkan, Academic)

Participants stated that adults tend to normalize abuse in sports environments. This situation causes the abuse experienced by children to be ignored and the abuse to continue:

“When parents come to support their children at matches, even though the coach shouts at the child unnecessarily and uses insulting words, the parents can say, ‘Hey, he’s the coach, he can do it’”.

(Ali, Coach)

It has been stated that female athletes face sexist prejudices, especially in male-dominated sports such as football. These prejudices limit female athletes’ opportunities to play sports and develop themselves and make them vulnerable to abuse:

“The football culture is terrible because the idea that women can’t play football is still prevalent. We cannot change this situation. No matter what we do, there is still this idea that football is a man’s game. “The question of whether a woman plays football is always asked. In addition to this, slang words are said from the stands”.

(Seda, Sportsman Athlete)

Participants stated that cultural norms and social pressure increase the risk of children being abused in sports environments. Especially in rural areas, social and cultural factors cause abuse to be normalized and ignored:

“I think your work will vary from big cities to rural areas. For example, we have coaches in the Antalya region. I mean, the parents are very friendly, very nice, and I like it very much. They cannot make eye contact with the trainer athlete in the Kars region. So there are also such factors, namely, whether they will reach out to the child and hug him/her, family events, regional events and climate events may also vary in this”.

(Yavuz, Coach)

#### 3.4.2. Policies and Procedures

Participants stated that one should be careful when choosing a coach. Choosing incompetent or abusive coaches increases the risk of children being abused:

“An athlete who is abused by his or her coach does not know what the correct behavior is. If children work with more enlightened, more visionary coaches for a long time, the athlete can understand what the correct behavior is. They may understand and perhaps give up this attitude. They can get rid of this pressure when they realize that they can express their thoughts more easily”.

(Sude, Athlete)

Participants stated that special organizations should be established to protect athletes’ rights. These organizations can provide support for athletes reporting abuse:

“I think athletes cannot say stop on their own because they are afraid of their coaches. I think such a private organization should be established. Just as there are awareness organizations in different areas, I think the same should be done for sports. “For example, team x but without revealing the player’s name… like revealing what he did”.

(Seda, Athlete)

It was emphasized that coaches should be made aware and behavioral norms should be established.

“Coaches also need to be made aware. It needs to be explained what is right and what is wrong. Because this is a two-way abuse, sometimes, like my experience, the coach can abuse a lot of athletes and normalize it. But otherwise the coach is very scared. “He is afraid of being investigated unfairly and complained about unfairly, so this time he cannot fulfill the requirements of sports”.

(Didem, Athlete)

Participants stated that information booklets should be published to prevent abuse in sports environments and to raise awareness:

“Perhaps if the ministry or federation publishes a booklet explaining the abuses specific to each sport, this can be delivered to athletes and parents through sports clubs”.

(Didem, Athlete)

It has been stated that criminal sanctions should be applied in cases of abuse. Holding coaches and other sport stakeholders accountable and punishing them in cases of abuse can contribute to preventing abuse:

“There needs to be sanctions. “Now, some coaches have lawsuits. There have been complaints about this, lawsuits have been filed, but generally, these are cases that are closed because there is insufficient evidence or because it is thought that there is no abuse”.

(Didem, Athlete)

## 4. Discussion

The findings of this study comprehensively reveal how child abuse occurs in sports environments within the framework of Ecological Systems Theory and what role environmental factors play in this process.

### 4.1. Types of Abuse

At the microsystem level, the types of abuse that children are exposed to in their direct interactions with coaches, teammates, and other athletes were examined. The types of abuse that occur at this level include physical, emotional, sexual abuse, and neglect. Physical abuse can be observed as contact and non-contact, as athletes are frequently dehydrated, encouraged to use doping and steroids, forced to continue playing when injured, and forced to train on unsuitable grounds. Additionally, physical violence is used by coaches and teammates in the form of objects being thrown or being hit with direct contact. Emotional abuse includes situations where athletes are subjected to physical actions such as throwing objects in anger or breaking doors, are forced to participate in matches and training with social and psychological pressure, are motivated through fear, and are kept under excessive control. Sexual abuse can range from non-contact abuse, such as inappropriate comments and behaviors and requests for inappropriate photographs, to contact abuse, such as inappropriate touching by coaches to athletes. Neglect includes situations such as neglecting the health and nutritional needs of athletes, not following weight control and diets, a lack of health services, and not providing emotional support. These types of abuse have serious negative effects on the physical and psychological health of athletes. These abuses, which occur at the microsystem level in interactions with coaches, teammates, and other athletes, form the basis of the negative experiences that children are exposed to in sports environments.

The findings of this study demonstrate important similarities and differences when compared to the existing literature on child abuse in sport settings. In the existing literature, it has been widely stated that children are exposed to physical abuse in sports environments [16,32,33]. While some researchers define physical abuse only as contact types of physical abuse, such as slapping, punching, pinching, pushing, and beating with a tool [33], other researchers include non-contact physical abuse, such as preventing a person from using the toilet or preventing a person from having the water or food they need. It has been addressed with an approach that includes preventing access to sleep, forcing overtraining that leads to the risk of injury, or forcing people to train while exhausted [1,32,34]. Similar findings were also detected in this study. However, unlike other studies, this study addresses contact and non-contact physical abuse types in more detail.

In terms of emotional abuse, the use of anger, humiliation, contempt, and excessive control towards athletes by coaches aand teammates has been widely covered in the literature [35]. We expanded the definition of emotional abuse by stating that coaches’ emotionally abusive behaviors consist of physical and verbal behaviors and denial of attention and support [36]. This study similarly detailed emotional abuse by coaches and teammates. For example, physical actions such as throwing objects and breaking doors in anger, social and psychological pressures such as forcing athletes to attend matches and training, motivating with fear and excessive control, and verbal abuse in the form of using sexist language, insults, and derogatory comments were observed. This study diversifies the examples of emotional abuse and reveals the different forms of such abuse in sports environments in more detail.

Non-contact and contact sexual abuse have been widely reported in the literature in sports environments. Such abuse includes sexual intercourse with an athlete, inappropriate sexual contact including genital penetration, making suggestive comments, jokes or gestures, sexual propositions, and showing pornographic images, videos, or other materials to an athlete [1,33,37,38,39]. This study also presents similar findings; abusive behaviors such as inappropriate comments, requests for photographs, and inappropriate touching were identified.

Neglect is defined in various dimensions in the literature. According to the definitions made by Stirling (2009), neglect can occur in different ways: physical (allowing alcohol and drug use, abandonment, and leaving injured), educational (preventing athletes from going to school), emotional (ignoring an athlete’s psychological health), and social (isolating athletes from their friends, parents, or romantic partners). In this study, definitions of neglect were further expanded with examples of health and nutrition and emotional neglect. While health and nutrition neglect involves depriving athletes of adequate and balanced nutrition opportunities, emotional neglect manifests as ignoring the emotional needs of athletes and depriving them of psychological support.

In this study, some participants described themselves as victims of abuse, while others stated that they had not had such an experience. While exposure to abuse causes some individuals to be more sensitive to abusive behaviors, it negatively affects the psychological state of others and may reduce the likelihood of reporting or talking about abuse experienced by others [40].

Coaches’ relationships with children significantly impact the risk of abuse. Authoritarian and controlling approaches and ignoring behaviors increase the risk of children being exposed to physical, emotional, and sexual abuse and neglect. In this study, the coaches’ authoritarian and controlling approaches created great pressure and stress on the athletes. Forcing children to continue playing, especially in the event of an injury, seriously endangers their physical health. This type of pressure can worsen children’s injuries and lead to long-term health problems. This finding is consistent with the negative effects that excessive pressure from coaches can have on athletes, as stated in the existing literature [7,35]. Similarly, in another study, some coaches with authoritarian behaviors use their informal power to manipulate athletes and sexually abuse them, leading to violence and sexual assault [40]. Coaches’ neglectful behavior toward children has serious negative effects on the physical and emotional health of athletes. Coaches’ ignoring of children’s nutritional and health needs is an example of neglect that negatively affects children’s physical capacities. As noted in the literature, neglect can leave deep scars on the physical and emotional health of athletes [1]. The authoritarian, controlling, and neglectful approaches displayed by coaches in their relationships with children increase the risk of children being exposed to various types of abuse. These findings are consistent with the effects of coach–child relationships on athletes reported in the literature and provide significant contributions to the knowledge in this field.

Relationships between teammates are an important environmental factor in the abuse experienced by children in sports settings. Peer bullying increases children’s risk of emotional and physical abuse. In this study, examples of peer bullying were identified, such as teammates using physical violence, making fun of clothing and outfits, and making derogatory comments. Prevalence studies in the literature show that the majority of interpersonal violence experienced by athletes is perpetrated by their peers [11,32]. For example, when Vertommen, Kampen, Schipper-van Veldhoven, Uzieblo, and Van Den Eede [11] examined retrospective accounts of 1785 adults who reported experiences of interpersonal violence in sports before the age of 18; they found that peers were the perpetrators in 82% of psychological violence cases, 45% of sexual violence cases, and 57% of physical violence cases. Stafford et al.’s [41] study highlights that children and adolescents can experience emotional harm from their peers in sporting environments. Similarly, research by Fasting et al., [42] “They coming from the countryside/rural areas”, reveals the existence of peer abuse during team initiation rituals or competitive stages. According to Jeckell et al. [43], peer abuse often results from misguided attempts to improve team harmony and unity. Therefore, in order to prevent peer bullying and abuse in sports environments, relationships and dynamics within teams need to be carefully managed.

### 4.2. Family and Coach Relationships

At the mesosystem level, interactions between microsystems were examined, and the effects of the relationship between the family and the coach on child abuse were addressed at this level. There are limited studies in the literature examining the relationship between family and coach [44,45,46]. According to Kerr and Stirling [45], some parents admitted to participating in the abuse by remaining silent and were therefore characterized as “silent bystanders.” Although the parents noticed the coach’s unethical behavior, they remained silent because of their respect for the coach and their emphasis on success in sports. This situation has contributed to the normalization of abuse. Stirling and Kerr [44] and Smits, Jacobs, and Knoppers [46] similarly stated that some athletes told their families about their coaches’ behavior, but the families did not intervene. This has led athletes to normalize these behaviors. Similar findings were obtained in our study. Families’ excessive trust in coaches and their lack of involvement in sports activities leaves children vulnerable to abuse. In addition, not allowing families to watch training causes the problems experienced by children to go unnoticed. This situation increases the risk of children being exposed to abuse. Our study emphasizes that it is important for families to manage their relationships with coaches more consciously and play a more active role in sports environments in preventing child abuse. These findings contribute to the literature emphasizing that families should take a more active role in sports environments.

### 4.3. Sports Clubs

At the exosystem level, environments that children do not interact with directly but that are indirectly affected by them are examined. Inadequate supervision and control mechanisms of coaches in sports clubs increase the risk of children being abused by malicious or incompetent coaches. In addition, the failure of clubs to take abuse cases seriously and to implement the necessary sanctions causes this problem to continue. Similarly, it is emphasized in the literature that sports clubs ignore abuse for fear of losing successful coaches [47]. Parent and Demers [48] observed that organizational managers were concerned that placing greater emphasis on harassment prevention programs would raise suspicions that harassment was occurring in their organizations.

Sports clubs and their managers need to develop more effective monitoring mechanisms to prevent abuse and take allegations of abuse seriously.

### 4.4. Social Environment and Cultural Factors

The findings of this study at the macrosystem level show how child abuse is shaped by environmental factors. In regions with disadvantaged socioeconomic conditions, children’s social development is not sufficiently supported. In particular, children from rural areas have fewer social opportunities, which can make them more vulnerable to negative behaviors such as bullying. As stated in the study, children from outer regions are ostracized and bullied by other children with more social opportunities. This finding is consistent with the literature showing that children’s social environment and socioeconomic status affect the risk of abuse [49,50]. Social harm, reduced social capital, lack of public awareness, and increasing socioeconomic inequalities can lead to children being abused and neglected or to unfair differences in their growth and development [50]. In addition, social isolation is considered one of the causes of child maltreatment [51]. The cultural values and social norms of the region where children are located greatly affect how child abuse is perceived and addressed in sports settings. Especially in regions with traditional social structures, the lack of trust and questioning in authority figures can cause the mistreatment and abuse that children encounter in sports settings to continue unnoticed. In rural or traditional communities, authority figures such as coaches and instructors who are considered powerful are respected without question, in line with the values of society. This situation allows coaches to establish greater authority over children and makes it difficult for families to intervene in cases of abuse. The statements of the participants also support this situation, showing that regional and cultural norms can directly affect the level of protection of children in sport settings. The upbringing, raising, and education processes of children are deeply shaped by the cultural codes and values of society. As stated by the World Health Organization [52], the attitudes of parents and other adults toward children are shaped by cultural codes. This situation becomes especially evident in traditional societies; great privileges are granted to those who are responsible for the education of children and are considered authority figures. In these societies, parents entrust their children to the care of these powerful authority figures with peace of mind, while the inadequacy or lack of child protection mechanisms is often ignored. A similar situation applies in sports environments [53]. Therefore, cultural and regional norms that affect the level of protection of children against abuse in sports environments, when combined with gender-based prejudices, further increase risk factors, especially for female athletes.

Our study indicated that female athletes face sexist prejudices, especially in male-dominated sports such as football. These prejudices limit female athletes’ opportunities to play sports and improve themselves, and they make them vulnerable to abuse. In the literature, gender stereotypes significantly affect societal attitudes toward women’s sports participation and field experiences [54,55]. Traditional sporting norms and gender-based prejudices prevalent in the sports environment have contributed to the formation of a culture in which sexual harassment and abuse are common [56]. Studies have found that girls are more likely to be abused than boys [57,58] and report such incidences more frequently [59]. This difference is related to men having difficulty reporting abuse and being reluctant to express their victimization due to social pressures [60]. In a cultural context where a man who reports abuse risk is deemed “not a real man,” men are less likely to express their victimization [61].

### 4.5. Necessary Policies and Procedures for Child Protection

At the macrosystem level, the importance of developing policies and procedures to reduce the risk of children being abused in sports environments is consistent with both the findings of this study and the existing literature. Participants made suggestions such as being meticulous in selecting coaches, establishing special organizations to protect athletes’ rights, publishing information booklets, and raising awareness among coaches. These recommendations are also supported in the literature [44,62]. This study also stated that child protection units should be established in sports clubs and penal sanctions should be applied in cases of abuse.

The International Safeguarding Children in Sport Working Group provides the International Safeguarding Children in Sport framework to ensure the protection of children in sports environments. These measures include developing policy and procedures for responding to protection concerns, providing advice and support, minimizing risks to children, establishing codes of conduct, managing recruitment, training, and communication processes, working with partners, and implementing monitoring and evaluation processes. Recent audits found that 125 organizations that have approved these safety measures work with more than 35 million children in total. This shows how comprehensive and effective child protection measures can be in sports [63].

## 5. Conclusions

This study examined child abuse in sports environments within the framework of Ecological Systems Theory and comprehensively addressed how environmental factors play a role in this process. The multi-layered approach provided by Ecological Systems Theory has allowed us to understand the dynamics of abuse not only in the context of individual or direct relationships but also in social and cultural contexts. Using this theoretical framework has shown that abuse is not only a one-to-one interaction at the microsystem level but also that broader environmental factors at the mesosystem, exosystem, and macrosystem levels are effective in this process. In this context, the findings demonstrate that the abuse children are exposed to in sports environments has a multidimensional structure and highlight the necessity of interventions at different levels in this process.

At the microsystem level, the types of abuse that children are exposed to in their relationships with people they interact with directly, such as coaches and teammates, were identified. Physical, emotional, and sexual abuse and neglect have been identified as common problems that children frequently encounter in sports environments. These types of abuse have been shown to have serious negative effects on children’s physical and psychological health. At the mesosystem level, the effects of family–coach relationships on child abuse were examined. Families’ excessive trust in coaches and the inadequate involvement in sports activities increase the risk of children being abused. The failure of families to participate in training makes it difficult to detect the problems experienced by children. These findings emphasize that families should take a more active role in sports environments. At the ecosystem level, the effects of management policies of sports clubs on child abuse were examined. The fact that coaches in sports clubs are not subject to adequate supervision and control mechanisms increases the risk of children being abused. The fact that sports clubs do not take abuse cases seriously and do not apply the necessary sanctions stands out as a major deficiency in terms of protecting children. At the macrosystem level, the effects of cultural norms and social environment on child abuse have been identified. The way children are raised, sexist prejudices, and limited social opportunities have been identified as important factors that increase the risk of children being abused in sports environments. Child protection policies and procedures play a critical role in preventing abuse and raising awareness.

One of the greatest contributions of this study is that it has revealed the multidimensional nature of child abuse in sports environments. The use of Ecological Systems Theory provided an understanding of this multidimensional structure and clearly demonstrated what levels of interventions were necessary to prevent abuse. Thus, it was emphasized that anti-abuse policies should be developed not only at the individual level but also at the social and cultural levels. This approach reveals that sports clubs, families, and society in general need to act in a more conscious and comprehensive way.

## 6. Practical Implications and Future Research Directions

The findings of this study highlight important practical steps and areas for research to improve child protection in sports settings. Comprehensive training is needed to help coaches set safe physical and emotional boundaries with children. Sports organizations should provide mandatory training that goes beyond technical skills to include social and psychological competencies such as ethical motivation, safe contact, and recognizing signs of distress in children. Increasing parental involvement and training is essential.

Parental overconfidence in coaches, combined with inadequate supervision, can increase the risk of abuse. Sports clubs can help parents become more proactive by providing programs that enable parents to monitor training, have regular meetings with coaches, and provide information on recognizing signs of abuse. Sports clubs need strong policies and procedures to prevent abuse. Clubs should strengthen their zero-tolerance approach to abuse by developing child protection policies that are in line with international standards. Policies should be developed that are responsive to regional and cultural contexts, taking into account the influence of cultural norms. In areas where social opportunities are limited or gender biases are strong, tailored interventions involving local community leaders may be more effective.

Future research could contribute to the development of culturally sensitive interventions by examining how abuse varies across cultural contexts. In addition, in-depth studies should be conducted to understand how parent–coach relationships affect the risk of abuse. Evaluating the impact of existing child protection programs and exploring digital tools that facilitate anonymous reporting of abuse by young athletes are also important areas of research.

## 7. Limitations

This study has several limitations. As a qualitative phenomenological study, the findings are specific to the experiences of athletes, coaches, and academics in sports environments in Turkey; therefore, they may not fully reflect experiences in other cultural or regional contexts. Additionally, the absence of athletes’ parents as respondents is an important limitation, as their participation could have contributed to understanding children’s experiences in sports environments from a parental perspective. The influence of the media was not directly investigated during the data collection phase. The media’s role in shaping societal attitudes toward sports, potentially normalizing or exacerbating certain behaviors, could be a relevant addition to the macrosystem level of analysis. Future research might include questions or measures that examine how media narratives impact perceptions and tolerance of abuse within sports settings.

## Data Availability

The data presented in this study are available on request from the corresponding author. The data are not publicly available due to privacy.
